# The Experience of Fertility Preservation in a Single Tertiary Center in Korea

**DOI:** 10.3389/fendo.2022.845051

**Published:** 2022-04-19

**Authors:** Yae Ji Choi, Yeon Hee Hong, Seongbeen Kim, Seul Ki Kim, Jung Ryeol Lee, Chang Suk Suh

**Affiliations:** ^1^ Department of Obstetrics and Gynecology, Seoul National University Bundang Hospital, Seongnam, South Korea; ^2^ Department of Obstetrics and Gynecology, Seoul National University College of Medicine, Seoul, South Korea; ^3^ Department of Surgical Oncology, Sheikh Khalifa Specialty Hospital, Ras Al Khaimah, United Arab Emirates

**Keywords:** embryo cyropreservation, fertility preservation, oncofertility, oocyte cryopreservation, ovarian tissue cryopreservation

## Abstract

**Objective:**

Oocyte (OC), embryo (EC), and ovarian tissue cryopreservation (OTC) are options for fertility preservation (FP) before going through gonadotoxic cancer treatment, or anticipated fertility decline in benign ovarian diseases, or for planned OC. The aim of this study is to report outcomes of FP in a single tertiary hospital in Korea.

**Methods:**

This is a retrospective study of OC, EC, and OTC cycles. All patients who visited or were referred to the infertility clinic at the Department of Obstetrics and Gynecology for the purpose of FP between 2010 and October 2021 were included.

**Results:**

A total of 564 controlled ovarian stimulation cycles were conducted in 416 women. Three hundred fifty-seven women underwent 494 OC cycles. Most patients were diagnosed with breast cancer (22.4%), followed by endometriomas (21.9%), and then by planned OC (20.7%). Cases of OC have increased over the years, peaking at 109 cycles in 2019 compared to one in 2010. Fifty-nine women underwent 70 EC cycles, and breast cancer (50.8%) was the most common indication. Repetitive OC and EC cycles were undergone in 92 and 9 women, respectively (mean number of repetition, 1.37 and 1.19 times in OC and EC, respectively), yielding a maximum number of 33 oocytes or 23 embryos being cryopreserved per patient. The utilization rate was 3.1% (11/357) in OC and 16.9% (10/59) in EC. Twenty-six women underwent OTC, and gynecologic cancer was the most common indication (9/26, 34.6%). One woman had the cryopreserved ovarian tissue retransplanted and successfully generated embryos.

**Conclusion:**

OC, EC, and OTC are possible options for preserving fertility, and these opportunities should be provided for women at risk of fertility decline or those who are eager to protect their future fertility. This is the first report on long-term FP outcomes in a single tertiary center in Korea. We expect that there will be more cases over the years and more women returning to use their gametes or embryos for pregnancy.

## Introduction

Fertility preservation (FP) is becoming an increasingly important field in women’s reproductive lives, and its demands are increasing rapidly. FP is considered in women before going through gonadotoxic cancer treatment or before anticipated fertility decline in the treatment of benign ovarian diseases.

Advancements in cancer treatment have resulted in decreased overall cancer mortality rates and increased long-term survival rate. The increased survival rate in reproductive-age women with cancer has, in turn, led to more women to have a chance to focus on long-term quality of life issues, such as motherhood. However, such cancer treatments, including chemotherapy and radiotherapy, inevitably risk compromising future fertility (1) due to its gonadotoxic effects. Chemotherapy drugs cause deoxyribonucleic acid (DNA) damage in oocytes that leads to chemotherapy-induced apoptosis and, in turn, to the irreversible decline of ovarian reserve, since the DNA damage usually cannot be repaired (2). Therefore, in female cancer patients, FP before cancer treatment is now considered essential rather than an option, and there is increased attention to FP not only by the medical staff but also by the patients themselves.

Planned oocyte cryopreservation (OC) or embryo cryopreservation (EC) is another indication of FP for women who are not married yet or want to postpone childbearing. In South Korea, the mean age at first live birth was delayed from 27.6 years in 1993 (3) to 31.9 years of age in 2018 (4), which is much higher than that in the United States where the mean age at first birth in 2018 was 26.9 years (5). Such growing trend of women delaying childbirth is associated with the pursuit of higher education, progression of professional career, and achievement of financial stability (4). Planned OC or EC could help avoid the natural decline in fertility while allowing women to maintain their childbearing decision-making for a longer duration (6).

The third, and the most overlooked, indication of FP is women who require surgical treatment of benign ovarian diseases, which anticipate the decrease of ovarian reserve. In particular, endometriosis of the ovary is the biggest threat to women’s fertility, since endometriosis itself can reduce the ovarian reserve by inflicting damage to the surrounding ovarian tissue (7) and ovarian cystectomy leads to a further decline of ovarian reserve (8). Bedoschi et al. (9) previously highlighted the importance of FP procedures in reproductive-age women at risk of impaired fertility related to endometriosis progression or endometriosis surgical treatment. A recent study by Hong et al. (10) reported that women with endometriosis had a significantly lower number of oocytes retrieved compared to infertile women without endometriosis, and therefore, the early timing of FP in women with such benign ovarian cysts is becoming of increased importance.

There are three main strategies of FP, which the present study focuses on: OC, EC, and ovarian tissue cryopreservation (OTC). Other strategies include ovarian suppression with gonadotropin-releasing hormone (GnRH) agonists and ovarian transposition. However, using GnRH agonists for ovarian protection is controversial (11) and not yet considered as an established method. Regarding this matter, previous studies have shown contradicting results and blinded or placebo-controlled randomized controlled studies are still lacking (12). The choice of the most appropriate method for FP depends on the type and timing of chemotherapy, type of cancer, patient’s age, and marital status (13). OC is an established technology for FP in postpubertal females, allows for increased control of future disposition of the oocytes, and may be considered the optimal strategy in women not in a stable relationship. EC is another strategy available for women who have a committed male partner, which leads to the highest likelihood of pregnancy success. OTC, although not as established as the former two strategies, is an acceptable FP method that the practice committee of the American Society for Reproductive Medicine (ASRM) stated is no longer considered experimental (14). It is the only method to preserve fertility in prepubertal girls or in those who cannot delay cancer treatment (15).

The aim of this study is to report outcomes of FP of over 10 years in a single tertiary hospital in Korea and to see its improvements. This study focuses not only on FP in cancer patients, and planned cryopreservation, but also on FP in patients prior to anticipated fertility decline in the treatment of benign ovarian diseases. This study hopes to improve clinicians’ awareness toward the various strategies of FP in any woman of reproductive age who needs or wants to preserve fertility.

## Materials and Methods

### Study Design

This single-center study retrospectively analyzed the FP outcomes of patients who were referred to or who visited the Fertility Center of Seoul National University Bundang Hospital between 2010 and October 2021. The indication for FP was one of the following: anticipated chemo- or radiation therapy, before scheduled ovarian surgery, or planned FP. Patients’ characteristics including age, body mass index (BMI), diagnosis of the disease, anti-Müllerian hormone (AMH) level, and indication of FP were included as study parameters. The mean number of oocytes retrieved and the number of cryopreserved oocytes or embryos from all the controlled ovarian stimulation (COS) cycles for each patient were recorded. The follow-up data of all the patients included were extracted from medical records.

### Ethics

The study received institutional review board approval (B-2110-714-104), and the informed consent was waived for a large number of patients with guaranteed anonymity.

### Controlled Ovarian Stimulation Protocols for Oocyte Cryopreservation or Embryo Cryopreservation

All patients who had COS cycles for oocyte retrieval carried out for FP were enrolled without any age limitations. Cases with cycle cancelations due to any reason were excluded, for example, follicular growth failure.

All cycles (n = 564) were conducted with gonadotropin-releasing hormone (GnRH) antagonist protocols. Cycles were initiated in the early follicular phase of the menstrual cycle in patients with benign diseases or those undergoing planned OC. For cancer patients, since it was important not to delay their planned cancer therapy, random start cycles were applied in most patients regardless of the early follicular phase timing. All patients underwent COS cycles with either recombinant follicle-stimulating hormone (rFSH, Gonal-F; Merck Serono, Geneva, Switzerland) or FSH combined with luteinizing hormone (LH) [human menopausal gonadotropin (hMG), Menopur; Ferring, Malmo, Sweden). The starting dose of gonadotropin was determined according to the age and ovarian reserve of the patients. When the leading follicle reached the size of 13–14 mm, a GnRH antagonist (Cetrolix, cetrotide 0.25 mg, Merck-Serono) was used to prevent premature LH surge. Final oocyte maturation was triggered by the injection of a recombinant human chorionic gonadotrophin (rhCG) (Ovidrel 250 µg, Merck-Serono) or 0.2 mg triptorelin (Decapeptyl, Ferring), or a combination of the two (250 μg of rhCG plus 0.2 mg of triptorelin), when two or more leading follicles measured 18 mm in diameter. In patients with hormone-dependent cancers (breast and endometrial cancer), an aromatase inhibitor (Femara 5 mg, Novartis, Switzerland) was coadministered with gonadotropin daily starting from the COS cycles to at least 7 days after oocyte retrieval. Transvaginal ultrasound-guided oocyte retrieval was carried out under sedation 36 h after triggering. Metaphase II (MII) oocytes were selected for OC. For EC, the additional process of intracytoplasmic sperm injection (ICSI) using sperm from the husband was carried out and cultured until cleavage stage. One patient had her embryos cultured until blastocyst stage. The embryos were classified as “good quality” for cleavage-stage embryos with grade 6B or more as assessed by the Steer method (16) and for blastocysts with grade 4BB or more for blastocysts as defined by Gardner and Schoolcraft (17).

The oocytes and embryos were vitrified using the Kitazato Vitrification Cryotop kit (Kitazato, Japan) according to the manufacturer’s protocol (18, 19). When warming, the vitrified oocytes or embryos in the CryoTop were immersed directly in 37°C warming solution. The warmed oocytes or embryos were transferred to sequential concentration of sucrose in basic medium.

### Method for Ovarian Tissue Cryopreservation

OTC was carried out in the following patients: (1) those who could not delay their cancer treatment, (2) prepubertal females, (3) those with primary ovarian insufficiency (POI) or impending POI (for example, Turner syndrome). Ovarian tissue retrieval was performed mainly by minimally invasive laparoscopic surgery. In all cases, the entire unilateral ovary was removed.

The OTC protocol has been previously described (20, 21). As soon as oophorectomy was performed, the removed ovaries were collected in Leibovitz’s (L-15; WelGene, Daegu, Korea) medium measuring 4°C and then delivered to the laboratory as quickly as possible. The medulla was removed from the cortex of the ovarian tissue. Both vitrification and slow-freezing methods were used to cryopreserve the ovarian tissues. For slow-freezing, the ovaries were sliced into fragments of 5 mm in length, 1–5 mm in width, and 1 mm in thickness. Then, the fragments were exposed to the freezing solution composed of Dulbecco’s phosphate-buffered saline (DPBS; Gibco, Paisley, UK), 20% synthetic serum substitute (SSS; Irvine Scientific, Santa Ana, CA, USA), 1.5 M dimethyl sulfoxide (DMSO; Sigma-Aldrich), and 0.1 M sucrose (Sigma-Aldrich) for 20 min at room temperature (RT). These fragments were then transferred into the 1.8-ml cryo-vials (Nunc, Roskilde, Denmark) prefilled with 800 μl of the freezing solution. Then, the cryo-vials were placed into an automated computer-controlled freezing system (Kryo-360; Planer, Sunbury-on-Thames, UK). The initial cooling rate was −2°C/min that was held at −7°C. Manual seeding was performed. After 10 min had passed, cooling was then continued at the rate of −0.3°C/min until −40°C, followed by the rate of −10°C/min until −150°C was reached. Then, the cryo-vials were plunged into liquid nitrogen (LN2). For vitrification, the ovaries were sliced into fragments of 10 mm in length and width and 1 mm in thickness. In majority of the cases, vitrification process was carried out by first equilibrating the fragments in 7.5% ethylene glycol (EG; Sigma-Aldrich, St. Louis, MO, USA) and 7.5% DMSO in DPBS with 20% SSS for 15 min at RT. This was then transported into a solution containing 15% EG and 15% DMSO in DPBS medium with 40% SSS for the next 10 min. The ovarian fragments were then placed in a minimum volume of solution onto the Kitazato vitrification plate (Cryotissue; Kitazato Corporation, Shizuoka, Japan). A sterile gauze was applied to the back of the plate to remove excess vitrification solution and then submerged directly into LN2 (22). The plate was then inserted into a protective container and placed into an LN2 storage tank. In cases with malignancy, one or two vials were biopsied as samples to confirm the absence of cancer metastasis.

### Analysis

The primary outcomes were cryopreserved oocytes or embryos and cumulative numbers of these parameters. Secondary outcomes were the total number of retrieved oocytes and the number of mature oocytes. Other parameters such as distribution by diagnosis, changes in annual treatment pattern, and usage rate of cryopreserved oocytes or embryos were also analyzed.

All statistical analyses were performed using the Statistical Package for the Social Sciences version 25.0 (SPSS Inc., Chicago, IL, USA). Continuous variables were demonstrated as median (minimum, maximum) values as indicated and categorical variables were demonstrated as “n (%)” in the tables.

## Results

### Oocyte Cryopreservation

**Figure 1:**
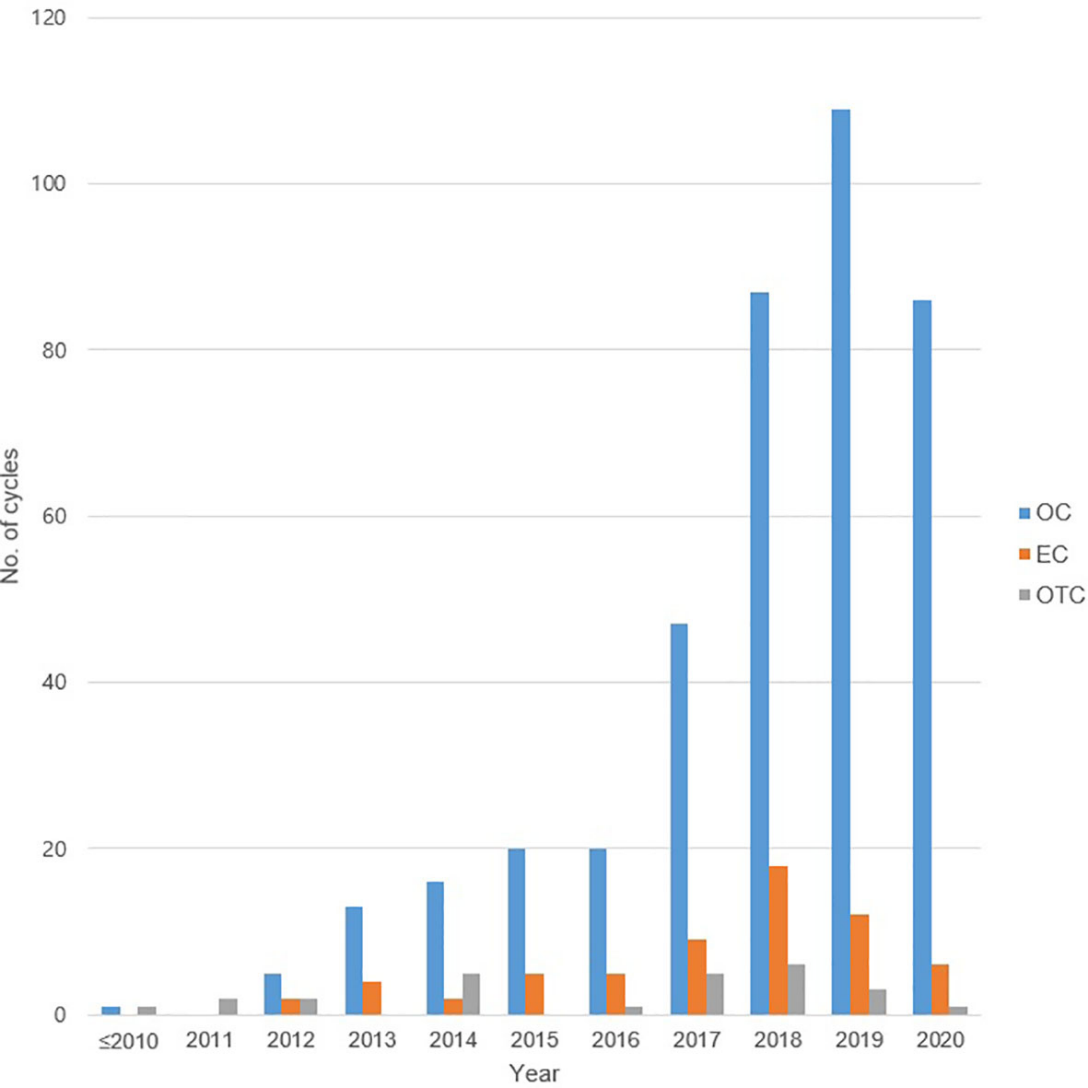
The number of total oocyte cryopreservation, embryo cryopreservation cycles, and ovarian tissue cryopreservation by year. OC, oocyte cryopreservation; EC, embryo cryopreservation; OTC, ovarian tissue cryopreservation.

A total of 564 COS cycles of OC or EC in 416 women were performed. Of this, 357 women underwent 494 OC cycles. The median age at the time of OC in all patients was younger than 35 years (31.0 years, range 12–50 years), and the median AMH level was 2.17 ng/ml (range 0.01–25.00 ng/ml). The most common indication for OC was breast cancer (n = 80, 22.4%), followed by ovarian endometrioma (n = 78, 21.9%), and then by planned OC (n = 74, 20.7%). The characteristics of the patients who underwent OC are shown in **Table 1A**. The staging of the malignancy is shown in **Supplementary Table S1**, and each was categorized into early- or advanced-stage disease in the same manner as previously described (23). The COS cycle outcomes of OC are recorded in **Table 1B**, and the median total number of oocytes retrieved was lowest in the group with endometrioma (5, range 1–25) and gynecologic cancers (5, range 0–23). Repetitive OC cycles were undergone in 92 women, and the mean number of repetitions was 1.37 times, yielding a maximum cumulative number of 33 oocytes being cryopreserved per patient. The number of COS cycles for OC showed an increasing trend over the years, peaking with 109 cycles in the year 2019 compared to one being performed between 2010 and 2011 (**Figure 1**).

**Table 1A:** The characteristics of patients who underwent oocyte cryopreservation (n = 357).

Diagnosis		No. of patients n (%)	Age (years)^a^	BMI (kg/m^2^)^a^	AMH (ng/ml)^a^
**Cancer**	**Breast**	80 (22.4)	34.0 (21–48)	21.6 (17.3–35.6)	3.28 (0.23–25.00)
	**Hematologic**	17 (4.8)	22.0 (16–33)	19.9 (16.3–26.7)	3.90 (0.04–13.40)
	**Gynecologic**	35 (9.8)	25.0 (13–40)	21.0 (17.1–27.6)	2.06 (0.28–15.57)
	**Gastrointestinal**	10 (2.8)	34.5 (24–38)	19.3 (15.8–22.6)	3.41 (1.11–5.94)
	Others^b^	20 (5.6)	26.5 (12–42)	21.0 (17.2–31.2)	4.72 (0.18–14.24)
**Benign**	**Endometrioma**	78 (21.9)	30.0 (18–41)	21.3 (16.8–29.0)	1.54 (0.12–7.72)
	Other ovarian cysts^c^	43 (12.0)	28.0 (19–41)	20.4 (17.0–32.1)	1.98 (0.27–13.50)
**Planned**		74 (20.7)	34.5 (13–50)	20.8 (13.5–34.1)	1.56 (0.01–17.90)

BMI, body mass index; AMH, anti-Müllerian hormone.

a All values are presented as median (minimum, maximum) values.

b Others include pancreatic cancer, bone sarcomas, malignant brain tumors, lung cancer, thymic cancer, thyroid cancer, and renal cancer.

c Other ovarian cysts include mature cystic teratoma, fibroma, thecoma, benign cystadenoma, and tubo-ovarian abscess.

**Table 1B:** The outcomes of the controlled ovarian stimulation cycles of oocyte cryopreservation (494 cycles).

Diagnosis		No. of cycles n (%)	No. of total oocytes retrieved (n)^a^	No. of initial MII retrieved (n)^a^	No. of oocytes cryopreserved (n)^a^
**Cancer**	**Breast**	92 (18.6)	8 (0–36)	4 (0–23)	5 (0–25)
	**Hematologic**	20 (4.0)	8 (0–29)	5 (0–20)	6 (0–22)
	**Gynecologic**	41 (8.3)	5 (0–23)	4 (0–20)	4 (0–20)
	**Gastrointestinal**	10 (2.0)	11 (5–40)	8 (3–24)	9 (4–33)
	Others^b^	23 (4.7)	9 (0–31)	6 (0–24)	8 (0–26)
**Benign**	**Endometriosis**	124 (25.1)	5 (1–25)	3 (0–19)	4 (0–20)
	Other ovarian cysts^c^	62 (12.6)	6 (1–23)	4 (0–14)	5 (1–17)
**Planned**		122 (24.7)	6 (0–43)	4 (0–28)	4 (0–31)

MII, metaphase of meiosis II, mature oocytes.

a All values are presented as median (minimum, maximum) values.

b Others include pancreatic cancer, bone sarcomas, malignant brain tumors, lung cancer, thymic cancer, thyroid cancer, and renal cancer.

c Other ovarian cysts include mature cystic teratoma, fibroma, thecoma, benign cystadenoma, and tubo-ovarian abscess.

Only 41 women out of the 357 who underwent OC (11.5%) were attempting to conceive, and the other 316 women were not currently attempting (88.5%). Out of the 41 women attempting, 13 women succeeded to conceive naturally, and 2 women succeeded *via* fresh *in vitro* fertilization (IVF). The utilization rate of the cryopreserved oocytes was 3.1% (11 out of 357 women), and one woman resulted in a successful pregnancy and a live birth. The main reasons for not attempting to become pregnant were due to their unmarried status (205 women), and 51 women were still receiving cancer treatment. Eight women who underwent OC died due to their original malignancy (**Table 2**). A total of 74 MII oocytes were thawed and showed a survival rate and fertilization rate of 87.8% (65/74) and 93.8% (61/65), respectively. The rate of good-quality embryo was 49.2% (30/61).

**Table 2:** Current status of patients who underwent oocyte cryopreservation and its utilization.

Not attempting to conceive yet (n = 316, 88.5%)	
Receiving cancer treatment	51
Death	8
Follow-up loss	11
Discarded due to patient request	10
Not married	205
No desire of conceiving although married	18
No oocytes retrieved in first place	13
**Attempting pregnancy (n = 41, 11.5%)**	
Natural pregnancy success	13
Natural pregnancy failure	13
Fresh IVF cycle success	2
Fresh IVF cycle failure	2
CO IVF cycle success	1^a^
CO IVF cycle failure	10^a,b^
Total	357

a Included in the calculation of utilization rate.

b Three of these women are still attempting IVF using the OC.

IVF, in vitro fertilization; CO, cryopreserved oocytes.

### Embryo Cryopreservation

Out of the 564 COS cycles, 59 women underwent 70 EC cycles. The characteristics and cycle outcomes of the patients who underwent EC are shown in **Tables 3A**, **3B**, respectively. Breast cancer was the most common indication comprising half of the patients (n = 30, 50.8%). The second common indications were gastrointestinal cancers and endometrioma (n = 7, 11.9%). Repetitive EC cycles were undergone in 9 women and the mean number of repetitions was 1.19 times, yielding a maximum cumulative number of 23 embryos cryopreserved. The utilization rate of cryopreserved embryos was 16.9% (10 out of 59 women) that was much higher compared to cryopreserved oocytes. A total of 17 (28.8%) women were attempting to conceive, and 4 of them had succeeded in achieving pregnancy through natural conception and 2 women succeeded *via* fresh IVF. Ten women attempted embryo transfer (ET) using cryopreserved embryos and 6 women succeeded in pregnancy, leading to a pregnancy rate of 60% (6 out of 10 women). Out of these 6 women, 5 of them had benign ovarian cysts and one had thyroid cancer. The most common reason for not attempting to become pregnant in women who underwent EC was since they were still receiving cancer treatment (23 women). Two women who underwent EC died due to their original malignancy (**Table 4**).

**Table 3A:** The characteristics of patients who underwent embryo cryopreservation (n = 59).

Diagnosis		No. of patients n (%)	Age (years)^a^	BMI (kg/m^2^)^a^	AMH (ng/ml)^a^
**Cancer**	**Breast**	30 (50.8)	34 (27–42)	21.7 (18.1–36.2)	2.85 (0.37–14.00)
	**Hematologic**	3 (5.1)	37 (28–38)	20.7 (17.9–23.6)	1.18 (1.03–2.23)
	**Gynecologic**	4 (6.8)	34 (29–41)	26.1 (19.7–30.5)	2.23 (1.33–3.71)
	**Gastrointestinal**	7 (11.9)	33 (26–39)	18.8 (12.4–22.8)	3.95 (2.08–5.35)
	Others^b^	3 (5.1)	36 (35–40)	21.3 (18.2–22.2)	2.03 (1.50–3.37)
**Benign**	**Endometrioma**	7 (11.9)	32 (29–38)	19.5 (17.7–31.6)	1.74 (0.13–3.66)
	Other benign cysts^c^	4 (6.8)	36 (36–37)	18.0 (17.7–22.1)	1.76 (0.84–3.13)
Others^d^		1 (1.6)	41	21.2	3.02

BMI, body mass index; AMH, anti-Müllerian hormone.

a All values are presented as median (minimum, maximum) values.

b Others include thyroid cancer and bone sarcoma.

c Other benign cysts include mature cystic teratoma and endodermal stromal tumor.

d Others include a patient who underwent EC prior to surgery for ovarian cancer, but final pathology revealed a uterine fibroid.

**Table 3B:** The outcomes of the controlled ovarian stimulation cycles of embryo cryopreservation (70 cycles).

Diagnosis		No. of cycles n (%)	No. of total oocytes retrieved (n)^a^	No. of initial MII retrieved (n)^a^	No. of embryo cryopreserved (n)^a^
**Cancer**	**Breast**	34 (48.6)	8 (0–28)	4 (0–19)	4 (0–23)
	**Hematologic**	3 (4.3)	9 (6–13)	7 (3–8)	6 (3–6)
	**Gynecologic**	6 (8.6)	6 (4–7)	2 (1–4)	2 (1–6)
	**Gastrointestinal**	8 (11.4)	14 (6–18)	9 (2–13)	9 (3–18)
	Others^b^	3 (4.3)	6 (6–11)	4 (1–8)	3 (2–9)
**Benign**	**Endometrioma**	10 (14.3)	6 (0–15)	4 (0–12)	3 (0–13)
	Other ovarian cysts^c^	5 (7.1)	5 (3–9)	5 (2–5)	3 (2–4)
Others^d^		1 (1.4)	5	1	5

MII, metaphase of meiosis II, mature oocytes.

a All values are presented as median (minimum, maximum) values.

b Others include thyroid cancer and bone sarcoma.

c Other benign cysts include mature cystic teratoma and endodermal stromal tumor.

d Others include a patient who underwent EC prior to surgery for ovarian cancer, but final pathology revealed a uterine fibroid.

**Table 4:** Current status of patients who underwent embryo cryopreservation and its utilization.

Not attempting to conceive yet (n = 42, 71.2%)	
Receiving cancer treatment	23
Death	2
Follow up loss	2
Discarded	1
No desire of conceiving	12
No cryopreserved embryo in first place	2
**Desiring pregnancy (n = 17, 28.8%)**	
Natural pregnancy success	4
Natural pregnancy failure	1
Fresh IVF cycle success	2
Fresh IVF cycle failure	0
CE IVF cycle success	6^a^
CE IVF cycle failure	4^a^
Total	59

IVF, in vitro fertilization; CE, cryopreserved embryos.

a Included in the calculation of utilization rate.

### Ovarian Tissue Cryopreservation

Twenty-six patients underwent OTC, and gynecologic cancer was the most common indication (n = 9, 34.6%), followed by hematologic cancer (n = 5, 19.2%) (**Table 5**). The median AMH level of the patients who underwent OTC was 2.59 (range 0.01–13.85) ng/ml. One woman had the cryopreserved ovarian tissue retransplanted that led to the first Korean case of a successful IVF-ET after transplantation of cryopreserved ovarian tissue (20). None of the patients had recurrent malignancy at follow-up during the study period. The mean duration of the cryopreserved ovarian tissue was 4.98 (± 2.85) years and the longest was stored for 12.5 years.

**Table 5:** The characteristics of patients who underwent ovarian tissue cryopreservation (n = 26).

Diagnosis	No. of patients n (%)	Age (years)^a^	BMI (kg/m^2^)^a^	AMH (ng/ml)^a^
Breast cancer	2 (7.7)	29 (27–32)	20.6 (19.7–21.4)	2.47 (1.98–2.95)
Hematologic cancer	5 (19.2)	19 (15–33)	18.4 (15.0–25.8)	4.59 (0.41–6.99)
Gynecologic cancer	9 (34.6)	32 (21–41)	21.1 (16.9–31.2)	3.98 (0.50–10.61)
Gastrointestinal cancer	1 (3.9)	31	20.0	–
Other malignancy^b^	5 (19.2)	12 (11–14)	17.3 (13.5–23.1)	4.91 (0.90–13.85)
Benign ovarian cyst^c^	1 (3.9)	32	28.0	3.41
Impending POI^d^	3 (11.5)	15 (12–26)	20.6 (19.3–33.8)	0.03 (0.01–0.08)

BMI, body mass index; AMH, anti-Müllerian hormone; POI, primary ovarian insufficiency.

a All values are presented as median (minimum, maximum) values.

b Other malignancy included malignant brain tumors and osteosarcoma.

c The patient underwent left salpingo-oophorectomy (LSO) for fibrothecoma.

d Two patients were diagnosed with Turner syndrome and one with idiopathic POI.

## Discussion

The present study showed the experience of FP in a single tertiary center during a relatively long period. For women facing imminent fertility decline or those who desired to postpone their childbearing plan, physicians must provide the patients appropriate counseling and FP opportunities. Furthermore, there were cumulative effects on the number of cryopreserved oocytes or embryos through repetitive COS. Hence, the repeat of COS, as long as it does not affect the treatment of the cancer or benign diseases, can lead to the optimal number of oocytes or embryos being cryopreserved.

The past 11 years of FP experience of this study showed an annual increasing trend of both OC and EC being performed. Other reports have shown a similar trend of increase in FP being performed in patients with cancer and planned OC. This is likely due to the increasing attention by physicians to the women’s desire of future pregnancy, after a stronger collaboration between their cancer center and fertility center (24), and also due to the increasing awareness of patients themselves. Such cooperation between oncologists and fertility specialists seems vital to provide the opportunity of FP in women who wish for future motherhood.

Nevertheless, FP in women before undergoing surgery for benign ovarian cyst has been overlooked. Even when taking care to preserve as much normal ovarian tissue as possible when performing ovarian cystectomy, it cannot be done perfectly and ovarian function decline is bound to be present after surgery and situations are worse with low basal AMH or bilateral cases (25). This is a much bigger issue in women with endometriosis, since endometriosis itself can reduce the ovarian reserve by inflicting damage to the surrounding ovarian tissue (7) and ovarian cystectomy leads to a further decline of ovarian reserve (8). A recent report suggested performing surgery after ovarian stimulation for FP in young women with endometriosis (26). With the importance of FP in this patient group being increasingly emphasized and the increased awareness at our center, we have shown that the highest percentage of OC being performed in women with benign ovarian diseases was for those with ovarian endometrioma.

The current report does not provide data regarding the rate of women who were offered FP in the first place. A report by Chung et al. (27) found that only 45.6% out of 457 clinicians in Hong Kong were aware of the importance to offer FP prior to anticancer treatment. Yee et al. (28) surveyed women in Canada who underwent planned OC from 2012 to 2018 and surprisingly found that only 12% of their respondents first learned about planned OC from a primary health care provider, while 37% first learned about it through the media. More clinicians need to be aware of their responsibility to provide women who are at risk of declining ovarian reserve with the chance of FP to improve their quality of life.

Our results showed an ongoing pregnancy rate of 9.1% per embryo transfer (1 out of 11) in OC cycles and a much higher ongoing pregnancy rate of 60% per embryo transfer (6 out of 10) in EC cycles. The ongoing pregnancy rate using cryopreserved oocytes in OC cycles was relatively low in our center. An explanation for such low pregnancy rate is due to the low number of women attempting pregnancy and an even lower utilization rate (only 41 women who underwent OC and 17 women who underwent EC were attempting to conceive). As we already mentioned, the utilization rate of cryopreserved oocytes was 3.1% (11 out of 357 women) with a similar value of 4.5% in another literature (24), and the utilization rate of cryopreserved embryos was 16.9% (10 out of 59 women). The most common reason for its non-utilization was their unmarried status in OC cycles, followed by the continuation of cancer therapy. For EC cycles, the most common non-utilization reason was that their cancer treatment was ongoing. Depending on the nature of the cancer, it could take a long time until anticancer therapy is complete and trial of conceiving can take place.

Regarding OTC, the total number of cases was much lower compared to EC and OC cycles, even though ASRM stated that OTC is no longer considered experimental (14). Only one patient returned to utilize her cryopreserved ovarian tissue, resulting in a utilization rate of 3.8%. Even though overall data of reproductive outcomes of OTC are still limited, current available data show that OTC should be offered to carefully selected patients that would lead to promising pregnancy outcomes. A report combining results of five major centers showed that the pregnancy rate was 29% in 111 patients who underwent transplant of cryopreserved ovarian tissue (29). Furthermore, the importance of OTC is being acknowledged in South Korea, and although relatively few, it is being performed continuously and steadily. In particular, there are patient groups to which OTC is the only option of FP possible, and therefore OTC is bound to be implemented as the main option alongside OC and EC in the near future.

The limitation of the present study is the retrospective nature and that the results of this single center cannot represent the whole of South Korea. In addition, the absolute number of OTC being performed is relatively low in South Korea, and live birth after its transplantation has not yet been achieved. On the other hand, a strong advantage of this report is that it is the first to provide data regarding three FP techniques of OC, EC, and OTC. Also, studies focusing on OC prior to anticipated ovarian function decline in benign ovarian diseases are rare, which should not be overlooked, especially in patients with endometriosis when infertility is of high concern.

In conclusion, centers performing FP must widen indications for OC, EC, and OTC, so that women are not deprived of a chance of pregnancy using their own gametes or embryos. We hope sharing data regarding outcomes of FP will improve awareness of both clinicians and women at risk of fertility potential decline and help identify the limits of the procedure for its further improvement in the future.

## Data Availability Statement

The original contributions presented in the study are included in the article/ **Supplementary Material** . Further inquiries can be directed to the corresponding author.

## Author Contributions

JRL, SKK, and CSS designed and conducted the research. YJC and SBK analyzed the data. YJC and YHH wrote the article. YHH and JRL reviewed the article. All authors read and approved the final article.

## Conflict of Interest

The authors declare that the research was conducted in the absence of any commercial or financial relationships that could be construed as a potential conflict of interest.

## Publisher’s Note

All claims expressed in this article are solely those of the authors and do not necessarily represent those of their affiliated organizations, or those of the publisher, the editors and the reviewers. Any product that may be evaluated in this article, or claim that may be made by its manufacturer, is not guaranteed or endorsed by the publisher.

## Abbreviations

AMH, anti-Müllerian hormone; ASRM, American Society of Reproductive Medicine; BMI, body mass index; CE, cryopreserved embryo; CO, cryopreserved oocytes; COS, controlled ovarian stimulation; DMSO, dimethyl sulfoxide; DNA, deoxyribonucleic acid; DPBS, Dulbecco’s phosphate-buffered saline; EC, embryo cryopreservation; EG, ethylene glycol; ET, embryo transfer; FP, fertility preservation; FSH, follicle-stimulating hormone; GnRH, gonadotropin-releasing hormone; hCG, human chorionic gonadotropin; ICSI, intracytoplasmic sperm injection; IVF, *in vitro* fertilization; LH, luteinizing hormone; LN2, liquid nitrogen; MII, metaphase II; OC, oocyte cryopreservation; OTC, ovarian tissue cryopreservation; POI, primary ovarian insufficiency; rFSH, recombinant follicle-stimulating hormone; rhCG, recombinant human chorionic gonadotropin; RT; room temperature; SPSS, Statistical Package for the Social Sciences; SSS, synthetic serum substitute.
