# Supplementary material for: The Experience of Fertility Preservation in a Single Tertiary Center in Korea
Source: Front Endocrinol (Lausanne). 2022 Apr 19;13:845051. doi: 10.3389/fendo.2022.845051 (PMC9062070; doi:10.3389/fendo.2022.845051)
Supplement: Supplementary file 1 [file Table_1.docx]

**Supplementary Table 1** The stage distribution of each cancer patients undergoing (A) oocyte cryopreservation, (B) embryo cryopreservation and (C) ovarian tissue cryopreservation

(A)

| **Cancer Type** | **Early-stage Disease** | **Advanced-stage Disease** | **Unknown** | **Total** |
| --- | --- | --- | --- | --- |
| Breast | 70 | 9 | 1 | 80 |
| Hematologic | 11 | 6 | 0 | 17 |
| Gynecologic | 25 | 10 | 0 | 35 |
| Gastrointestinal | 2 | 8 | 0 | 10 |
| Others | 3 | 15 | 2 | 20 |

(B)

| **Cancer Type** | **Early-stage Disease** | **Advanced-stage Disease** | **Unknown** | **Total** |
| --- | --- | --- | --- | --- |
| Breast | 27 | 3 | 0 | 30 |
| Hematologic | 3 | 0 | 0 | 3 |
| Gynecologic | 4 | 0 | 0 | 4 |
| Gastrointestinal | 3 | 3 | 1 | 7 |
| Others | 1 | 1 | 1 | 3 |

(C)

| **Cancer Type** | **Early-stage Disease** | **Advanced-stage Disease** | **Unknown** | **Total** |
| --- | --- | --- | --- | --- |
| Breast | 2 | 0 | 0 | 2 |
| Hematologic | 2 | 3 | 0 | 5 |
| Gynecologic | 7 | 2 | 0 | 9 |
| Gastrointestinal | 0 | 1 | 0 | 1 |
| Others | 4 | 1 | 0 | 5 |
